# Determinants of arterial stiffness progression in a Han-Chinese population in Taiwan: a 4-year longitudinal follow-up

**DOI:** 10.1186/s12872-015-0093-2

**Published:** 2015-09-16

**Authors:** Lien-Ying Lin, Yi-Chu Liao, Hsiu-Fen Lin, Yu-Shan Lee, Reuy-Tay Lin, Chung Y. Hsu, Suh-Hang H. Juo

**Affiliations:** 1 Department of Neurology, Neurological Institute, Taichung Veterans General Hospital, 1650 Taiwan Boulevard Sect. 4, Taichung, 407 Taiwan, ROC; 2 Department of Neurology, Taipei Veterans General Hospital, No.201, Sec. 2, Shipai Rd., Beitou District, Taipei, 112 Taiwan, ROC; 3 Department of Neurology, School of Medicine, National Yang-Ming University, No.155, Sec.2, Linong Street, Taipei, 112 Taiwan, ROC; 4 Department of Neurology, Kaohsiung Medical University Hospital, No.100, Tzyou 1st Road Kaohsiung, Kaohsiung, 807 Taiwan, ROC; 5 Department of Medical Research, Kaohsiung Medical University Hospital, No.100, Tzyou 1st Road Kaohsiung, Kaohsiung, 807 Taiwan, ROC; 6 Department of Neurology, College of Medicine, Kaohsiung Medical University, No.100, Shih-Chuan 1st Road, Kaohsiung, 807 Taiwan, ROC; 7 Department of Medical Genetics, College of Medicine, Kaohsiung Medical University, No.100, Shih-Chuan 1st Road, Kaohsiung, 807 Taiwan, ROC; 8 Institute of Clinical Medicine, China Medical University, No.91, Hsueh-Shih Road, Taichung, 404 Taiwan, ROC; 9 Department of Neurology, China Medical University Hospital, 2 Yude Road, Taichung, 404 Taiwan, ROC

**Keywords:** Arterial stiffness, Pulse wave velocity, Progression rate, Blood pressure, Obesity

## Abstract

**Background:**

Arterial stiffness predicts the future risk of macro- and micro-vascular diseases. Only a few studies have reported longitudinal changes. The present study aimed to investigate the progression rate of arterial stiffness and the factors influencing stiffness progression in a Han Chinese population residing in Taiwan.

**Methods:**

The pulse wave velocity (PWV), elasticity modulus (Ep) and arterial stiffness index (β) of the common carotid artery were measured in 577 stroke- and myocardial infarction-free subjects at baseline and after an average interval of 4.2 ± 0.8 years. Stepwise multivariate linear regression was conducted to elucidate the predictors of stiffness progression.

**Results:**

For both baseline and follow-up data, men had significantly higher values of PWV, Ep and β in comparison to women. The progression rates of PWV, Ep and β were faster in men, but the difference was not statistically significant (ΔPWV = 0.20 ± 0.20 and 0.18 ± 0.20 m/s/yr; ΔEp = 8.17 ± 8.65 and 6.98 ± 8.26 kPa/yr; Δβ = 0.70 ± 0.64 and 0.67 ± 0.56 for men and women, respectively). In the multivariate regression analyses, age, baseline stiffness parameters, baseline mean arterial pressure (MAP), baseline body mass index (BMI) and changes in MAP (ΔMAP) were independent predictors of PWV and Ep progression. There was an inverse correlation between the stiffness parameters at baseline and their progression rate (correlation coefficient (r) = −0.12 to −0.33, *p* = 0.032–1.6 × 10^−16^). Changes in MAP (ΔMAP) rather than baseline MAP were more strongly associated with PWV progression (*p* = 8.5 × 10^−24^ and 1.9 × 10^−5^ for ΔMAP and baseline MAP, respectively). Sex-specific analyses disclosed that baseline BMI and changes in BMI (ΔBMI) were significantly associated with stiffness progression in men (*p* = 0.010–0.026), but not in women.

**Conclusions:**

Aging and elevated blood pressure at baseline and during follow-up were the major determinants of stiffness progression in the Han Chinese population. For men, increased baseline BMI and changes in BMI were additional risk factors.

**Electronic supplementary material:**

The online version of this article (doi:10.1186/s12872-015-0093-2) contains supplementary material, which is available to authorized users.

## Background

Arterial stiffness, which is caused by the loss of normal elastin and the increase of abnormal collagen, is one of the earliest functional changes in the vascular aging process [1]. Previous studies have shown that increased arterial stiffness is strongly associated with atherosclerosis [2]. Arterial stiffness predicts future risk of coronary heart disease, stroke, and cardiovascular mortality in high-risk and general populations [3–5]. In addition, arterial stiffness contributes to the hypertrophy and remodeling of microcirculation [6], which leads to microvascular diseases, such as diabetic retinopathy and lacunar infarction [7, 8]. Elucidating the determinants of arterial stiffness could pave the way toward the management and prevention of both macro- and micro-vascular diseases.

The established risk factors for arterial stiffening include aging, elevated blood pressure (BP), impaired glucose metabolism, hyperlipidemia, and increased body mass index (BMI) [9, 10]. Most studies evaluated stiffness parameters in a cross-sectional manner; only a few large-scale cohort studies reported the longitudinal changes of stiffness [11–15]. The majority of the participants in these longitudinal studies were Caucasians. The MultiEthnic Study of Atherosclerosis enrolled only 308 Chinese, and according to MESA, Chinese subjects had significantly worse profiles of arterial stiffness at baseline, but the rate of arterial stiffening in Chinese subjects during follow-up was similar to the rates in other ethnic groups [13]. Only one longitudinal study investigated the association between metabolic syndrome and brachial-ankle PWV (baPWV) in a Taiwanese population [16], but baPWV could not faithfully denote central arterial stiffness [17]. The features of the progression of central arterial stiffness in non-white populations remain unclear.

This longitudinal study aims to follow-up on the progression rate of arteriosclerosis in the Chinese population residing in Taiwan. The present study reported the progression rate of arterial stiffness and the factors influencing the progression of stiffness in this cohort. In addition, sex-specific factors in relation to stiffness progression were explored.

## Methods

### Study subjects

The study participants were stroke- and myocardial infarction-free individuals recruited from the community population through an advertisement posted at the Kaohsiung Medical University Hospital (KMUH) [18]. Carotid ultrasonography was performed in each participant for the measurement of arterial stiffness. From November 2008 through March 2014, we contacted 579 subjects who completed baseline assessments between August 2006 and August 2010. Two participants (0.3 %) were lost to follow-up, and 577 persons received a second carotid ultrasonographic study.

Each participant filled out a self-administered questionnaire, which included demographic information, previous medical histories and smoking habits. BMI was calculated as weight (kg)/ height squared (m^2^). BP was acquired using a calibrated standard sphygmomanometer (Omron; Vernon Hills, Illinois) after resting in the sitting position for at least 5 min. The average value from two measurements was used. Plasma concentrations of total cholesterol (TC), high density lipoprotein-cholesterol (HDL-C), fasting blood sugar (FBS), and triglyceride (TG) were measured using standardized enzymatic procedures (Boehringer Mannheim, Germany). The study was approved by the Institutional Review Board of KMUH and written informed consent was provided by each participant.

### Measurement of arterial stiffness

Carotid stiffness was measured by an ultrasound with an echo-tracking system (SSD-5500, Aloka, Tokyo, Japan) equipped with a 3–12-MHz linear array transducer and a vessel wall movement detector. Measurements were performed in the right common carotid artery (CCA) 2 cm before the carotid bulb, and a longitudinal section of the vessel was obtained in the B-mode. In the M-mode, the vessel movement detector system registered at least 10 consecutive cardiac cycles and the subsequent changes in arterial diameter. Brachial BP was taken with a semi-automated recorder before and after each ultrasound examination and was averaged. This procedure was repeated three times, and the average data were used for analyses [18, 19]. Three carotid stiffness parameters were calculated automatically:

1. Arterial stiffness index (*β*) = Ln (SBP / DBP)/[(D_s_ − D_d_)/ D_d_] (SBP and DBP are the systolic and diastolic BP; Ds and Dd are the systolic and diastolic intra-luminal CCA diameters);
2. Elasticity modulus (Ep) = (SBP − DBP)/[(Ds − Dd)/Dd]; and
3. One point pulse wave velocity (PWV) = √ (*β*DBP/2*ρ*), where ρ is the constant of blood density (1050 kg/m^3^).

PWV was conventionally measured using a “two-point” method, while the Aloka ultrasound system provided a valid, one-point measurement of PWV [20]. The intra-reader correlation coefficient was 0.98 in β and Ep and 0.92 in PWV.

### Definition of risk factors

The conventional risk factors for arterial stiffness include diabetes, hypertension, hyperlipidemia, obesity and smoking habit. A subject was defined as having diabetes if he/she had FBS ≥126 mg/dl or was on hypoglycemic medications. A subject was defined as having hyperlipidemia if he/she had serum levels of TC ≥ 240 mg/dl or was on hypolipidemic agents. Hypertension was defined as BP ≥ 140/90 mmHg or was on anti-hypertensive medication. Obesity was defined as BMI ≥ 25 kg/m^2^ according to the Asian criteria proposed by International Association for the Study of Obesity and the International Obesity Task Force [21]. Because conventional risk factors might be modified during follow-up, changes in mean arterial pressure (ΔMAP), lipid profiles (ΔTC, ΔTG and ΔHDL-C), FBS (ΔFBS) and BMI (ΔBMI) were used to quantitatively measure the improvement of these risk factors. The change in each risk factor parameter was calculated by subtracting the value measured at follow-up by the corresponding value upon enrollment.

### Statistical analyses

Statistical analyses were performed using SPSS 19.0 (SPSS Inc., Chicago, IL, USA). A two-tailed *p* value < 0.05 was considered statistically significant. Data were presented as the means ± standard deviation (SD) or proportions. The chi-squared test was used for categorical data, while Student’s *t*-test was used for continuous data. Serum TG levels were log-transformed to approximate the normal distribution. The annual progression rate of arterial stiffness was defined as the difference of the individual stiffness parameter divided by the follow-up interval between two ultrasonography examinations. Pearson’s correlation was used to evaluate the relationship between stiffness progression rates and quantitative risk factors at baseline, as well as the relationship between stiffness progression rates and changes in each risk factor parameter during follow-up.

Stepwise multivariate linear regression was conducted to investigate the predictive effect of age, baseline risk factors and changes in quantitative risk factors for stiffness progression. Risk factors associated with progression rates with a *p* value < 0.10 in the univariate analysis were included in the multivariate regression model. All the statistical analyses were repeated in sex-specific analyses.

## Results

### Demographic features

The demographic characteristics of the study subjects are shown in Table 1. The average age at enrollment was 54.5 ± 9.2 years, and men accounted for 38.8 % of the study participants. The average interval between two ultrasonography examinations was 4.2 ± 0.8 years. At baseline, men had a higher proportion of diabetes and smoking habit and were more obese than women. Men had higher values of FBS, TG and BMI, while women had higher values of HDL-C and TC. The above patterns remained in the second measure.

**Table 1:** Demographic features of study participants

% or mean ± SD	All (*N* = 577)		Women (*N* = 353)		Men (*N* = 224)	
Follow-up duration (y)	4.2 ± 0.8		4.3 ± 0.8		4.2 ± 0.8	
	Baseline	Follow-up	Baseline	Follow-up	Baseline	Follow-up
Age (y)	54.5 ± 9.2	58.7 ± 9.2	54.0 ± 9.2	58.2 ± 9.2	55.2 ± 9.3	59.4 ± 9.3
Hypertension (%)	29.5	39.7	28.9	38.0	30.4	42.4
Diabetes (%)	8.5	13.0	4.9	9.6	14.3 **	18.3 **
Hyperlipidemia (%)	21.7	40.6	20.9	40.5	23.3	40.6
Obesity (BMI ≥ 25 kg/m^2^) (%)	31.0	41.6	27.6	34.6	46.0 **	54.8 **
Ex- and current smoker (%)	16.3	19.6	3.4	4.0	40.2 **	44.2 **
MAP (mmHg)	88.8 ± 10.3	85.2 ± 9.6	88.2 ± 10.7	83.9 ± 9.6	89.6 ± 9.6	87.3 ± 9.2 **
FBS (mg/dL)	103.7 ± 23.2	99.5 ± 22.0	101.2 ± 20.4	98.0 ± 23.5	107.7 ± 26.6 **	101.7 ± 19.2
TC (mg/dL)	200.5 ± 37.4	206.3 ± 36.0	203.8 ± 35.8	209.3 ± 35.3	195.2 ± 39.3 *	201.7 ± 36.7 *
TG (mg/dL)	120.8 ± 72.1	120.1 ± 71.6	112.6 ± 66.5	114.2 ± 70.0	133.63 ± 78.76 **	129.5 ± 73.4 *
HDL-C (mg/dL)	56.4 ± 15.0	57.6 ± 14.5	60.7 ± 15.7	61.9 ± 14.7	49.6 ± 10.9 **	50.8 ± 11.2 **
BMI (kg/m^2^)	24.3 ± 3.2	24.7 ± 3.4	23.7 ± 3.1	24.0 ± 3.3	25.3 ± 3.1 **	25.7 ± 3.3 **
Arterial stiffness						
PWV (m/s)	6.1 ± 1.1	6.9 ± 1.2	6.0 ± 1.1	6.7 ± 1.1	6.3 ± 1.2 **	7.2 ± 1.3 **
EP (kPa)	104.7 ± 43.3	135.8 ± 53.3	100.8 ± 41.1	130.2 ± 48.6	110.9 ± 46.1 **	144.7 ± 59.0 **
β	8.0 ± 2.9	10.9 ± 3.8	7.7 ± 2.6	10.6 ± 3.5	8.4 ± 3.2 **	11.4 ± 4.2 *
Progression rate of arterial stiffness						
ΔPWV/yr (m/s/yr)	0.19 ± 0.20		0.18 ± 0.20		0.20 ± 0.20	
ΔEP/yr (kPa/yr)	7.44 ± 8.43		6.98 ± 8.26		8.17 ± 8.65	
Δβ/yr	0.68 ± 0.59		0.67 ± 0.56		0.70 ± 0.64	

*BMI* body mass index, *FBS* fasting blood sugar, *MAP* mean arterial pressure, *TG* triglyceride, *TC* total cholesterol, *HDL-C* high-density lipoprotein cholesterol

*P <0.05, **P < 0.01, in comparison between men and women using Student’s *t* test or Chi-squared test

**Fig. 1:**
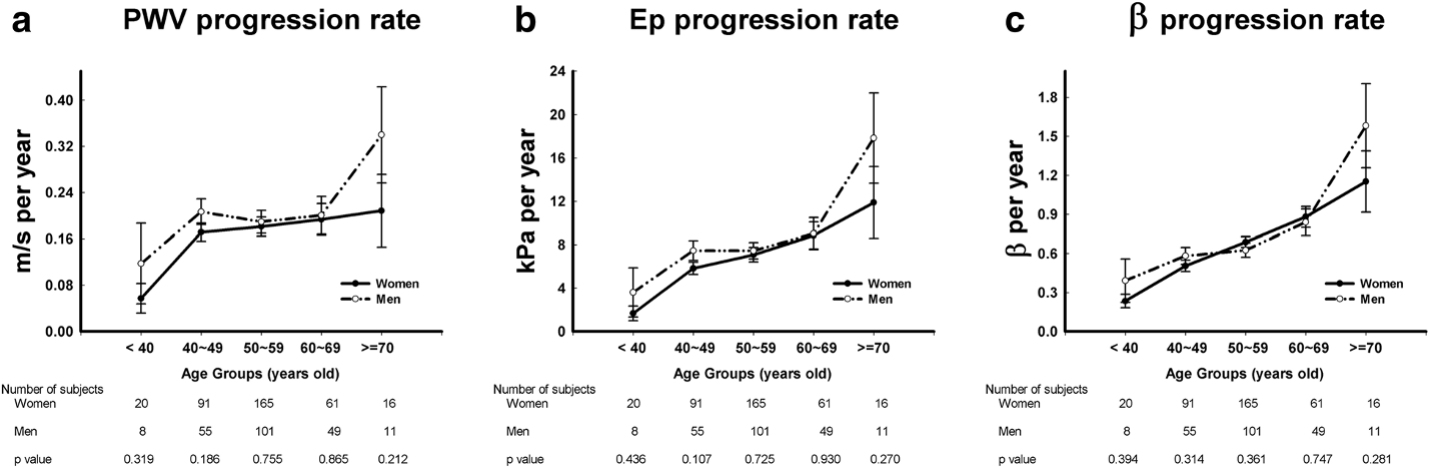
Sex-specific progression rates of (**a**) PWV (**b**) Ep and (**c**) β in different age groups

For both baseline and follow-up data, men had significantly higher values of PWV, Ep and β in comparison to women (Table 1). The progression rates of PWV, Ep and β were faster in men, but the difference was not statistically significant (Fig. 1). The three stiffness parameters were strongly correlated with each other, and there was an inverse correlation between the stiffness parameters at baseline and their progression rates (Table 2). For example, a higher value of PWV at baseline (i.e., stiffer arteries) was associated with a slower progression rate of PWV at follow-up.

**Table 2:** Pearson’s correlation coefficient (r) between risk factors and progression rate of arterial stiffness

	Stiffness parameters at baseline			Progression rate of stiffness parameters		
	PWV	Ep	β	ΔPWV/yr	ΔEP/yr	Δβ/yr
PWV	--	0.97 **	0.93 **	- 0.33 **	- 0.18 **	- 0.10 *
EP	--	--	0.95 **	- 0.34 **	- 0.19 **	- 0.09 *
β	--	--	--	- 0.30 **	- 0.17 **	- 0.12 *
MAP	0.44 **	0.41 **	0.12 **	- 0.17 **	- 0.08 *	0.04
FBS	0.09 *	0.09 *	0.09 *	0.04	0.05	0.06
TG	0.26 **	0.21 **	0.16 **	0.02	0.03	0.07
HDL-C	- 0.21 **	- 0.21 **	- 0.17 **	0.04	0.02	- 0.02
BMI	0.30 *	0.26 **	0.21 **	0.03	0.08 *	0.10 *
ΔMAP	--	--	--	0.42 **	0.38 **	- 0.004
ΔTC	--	--	--	0.13 **	0.11 *	0.06
ΔTG	--	--	--	0.08 *	0.08 *	0.04
ΔBMI	--	--	--	0.08 *	0.08 *	0.05

Number in the table is partial Pearson’s correlation coefficient (r) with adjustment of age and sex

ΔMAP, ΔFBS, ΔTC, ΔTG, ΔHDL-C, and ΔBMI are calculated by subtracting the values at follow-ups by the corresponding values at baseline

TG was log-transformed to approximate the normal distribution. Variables (TC, ΔHDL-C, and ΔFBS) not associated with any stiffness parameter were not shown

* = *p* < 0.10, ** = *p* < 0.01

### Cardiovascular risk factors and stiffness progression

Conventional risk factors including diabetes, hypertension and obesity were associated with more advanced stiffness at baseline (Additional file 1: Table S1). However, these baseline risk factors did not significantly predict progression rate in any of the three parameters. Each risk factor was associated with faster progression rate in men than in women, but the differences did not reach any statistical significance (Additional file 1: Table S2).

We further used Pearson’s correlation to evaluate the relationship between quantitative risk factors and stiffness progression rate with the adjustment of age and sex (Table 2). Only baseline MAP and BMI were correlated to the stiffness progression rates. The changes in MAP (ΔMAP) and TC (ΔTC) during the follow-up period were significantly correlated with the progression rates of PWV and Ep. Furthermore, changes in BMI (ΔBMI) and TG (ΔTG) were associated with the progression rates of PWV and Ep with borderline *p* values < 0.10.

### Independent predictors of stiffness progression

Stepwise multivariate regression analyses were conducted to identify the independent predictors of stiffness progression. Only risk factors that were related to the progression rates in the initial analyses with *p* values < 0.10 were included. Age, baseline stiffness parameters, baseline MAP, baseline BMI and changes in MAP (ΔMAP) were found to be independent predictors of PWV and Ep progression (Table 3). In fact, ΔMAP was more strongly associated with PWV progression than baseline MAP. Similar findings were also noted for the rate of Ep progression but not for the rate of β progression. Although changes in plasma TC and TG levels were associated with stiffness progression in the univariate analysis, the significance disappeared after adjustment for other risk factors. Baseline BMI and change in BMI (ΔBMI) were significantly associated with a faster progression of PWV, Ep and β in men but not in women.

**Table 3:** Predictors of stiffness progression rate by multivariate regression analysis

Covariates	All (*N* = 577)		Women (*N* = 353)		Men (*N* = 224)	
PWV progression	Coefficient (SE)	*p* value	Coefficient (SE)	*p* value	Coefficient (SE)	*p* value
Age	0.007 (0.001)	<0.001	0.008 (0.001)	<0.001	0.007 (0.002)	<0.001
Male	- 0.008 (0.016)	0.604	--	--	--	--
Baseline PWV (m/s)	- 0.075 (0.008)	<0.001	- 0.086 (0.010)	<0.001	- 0.059 (0.012)	<0.001
MAP (mmHg)	0.004 (0.001)	<0.001	0.006 (0.001)	<0.001	--	--
BMI (kg/m^2^)	0.008 (0.002)	0.002	--	--	0.012 (0.005)	0.011
ΔMAP (mmHg)	0.010 (0.001)	<0.001	0.010 (0.001)	<0.001	0.008 (0.002)	<0.001
ΔBMI (kg/m^2^)			--	--	0.024 (0.010)	0.013
Ep progression	Coefficient (SE)	*p* value	Coefficient (SE)	*p* value	Coefficient (SE)	*p* value
Age (y)	0.369 (0.039)	<0.001	0.412 (0.049)	<0.001	0.349 (0.066)	<0.001
Male	- 0.748 (0.679)	0.271	--	--	--	--
Baseline Ep (kPa)	- 0.060 (0.009)	<0.001	- 0.072 (0.012)	<0.001	- 0.050 (0.015)	0.001
MAP (mmHg)	0.194 (0.042)	<0.001	0.251 (0.050)	<0.001	0.163 (0.074)	0.028
BMI (kg/m2)	0.327 (0.108)	0.003	--	--	0.473 (0.191)	0.014
ΔMAP (mmHg)	0.401 (0.041)	<0.001	0.420 (0.049)	<0.001	0.360 (0.074)	<0.001
ΔBMI (kg/m2)	--	--	--	--	0.916 (0.409)	0.026
β progression	Coefficient (SE)	*p* value	Coefficient (SE)	*p* value	Coefficient (SE)	*p* value
Age (y)	0.028 (0.003)	<0.001	0.030 (0.004)	<0.001	0.028 (0.005)	<0.001
Male	- 0.052 (0.052)	0.318	--	--	--	--
Baseline β	- 0.041 (0.010)	<0.001	- 0.042 (0.012)	0.001	- 0.045 (0.015)	0.004
MAP (mmHg)	--	--	--	--		
BMI (kg/m2)	0.024 (0.008)	0.003	--	--	0.038 (0.015)	0.010
ΔMAP (mmHg)	--	--	--	--	--	--
ΔBMI (kg/m2)	--	--	--	--	0.078 (0.032)	0.015

Covariates included in the stepwise multivariate regression model were age at baseline, sex, baseline stiffness parameters, MAP at baseline, BMI at baseline, ΔMAP, ΔTC, ΔTG and ΔBMI. Only variates with significant *p* value were retained in the final model

**Fig. 2:**
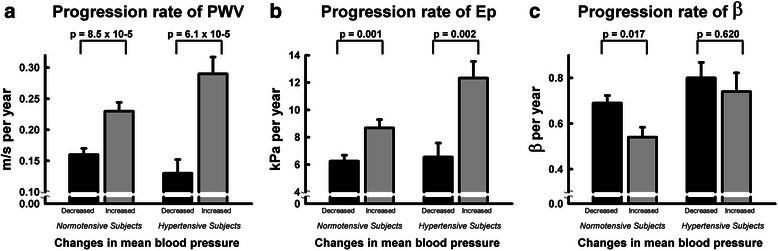
Fair-controlled blood pressure during follow-up period is associated with slower progression rate of (**a**) PWV (**b**) Ep and (**c**) β in hypertensive and normotensive subjects

To further delineate the relationship between ΔMAP and stiffness progression, study subjects were divided into two groups based on the increase or decrease of MAP during the follow-up period. Figure 2 showed that an increase in MAP (ΔMAP > 0 mmHg) during the follow-up period was significantly associated with a more rapid progression rate of PWV in both hypertensive subjects and in normotensive subjects. A similar pattern was also observed in Ep but not in β. More specifically, baseline MAP and ΔMAP were not related to β progression.

## Discussion

The present study reported the progression rate of carotid stiffness in a Chinese population during an average follow-up of 4.1 years. Although men tended to have faster progression of all of the stiffness parameters relative to women, the difference did not reach statistical significance. Conventional risk factors including hypertension, diabetes, obesity, and serum lipid profile were correlated with a stiffer artery at baseline, but they were not associated with any stiffness progression rates. The major determinants of the stiffness progression rate were (1) age, (2) severity of stiffness at baseline, (3) baseline MAP and ΔMAP, and (4) baseline BMI. Additionally, gain of BMI during the follow-up period was significantly associated with a faster progression in men, but not in women.

In this study, three stiffness parameters (Ep, β, and PWV) were used to measure the arterial stiffness. Although the three parameters are well correlated, they reflect different aspects of vascular property. PWV is generally considered to be the gold standard measure of systemic arterial stiffness [22, 23], whereas Ep and β are determinants of local vessel wall elasticity [22, 24]. Ep is vulnerable to the effect of pulse pressure, while β is the natural logarithm of SBP and DBP ratio and thus is relatively independent of BP [25, 26]. That explains why MBP was significantly correlated with PWV and Ep, but not associated with β in the current study. To be noticed, one-point carotid PWV instead of carotid-femoral PWV was measured by ultrasound with an echo-tracking system in the present study. The validity of one-point carotid PWV was supported by literature that demonstrated a good correlation between one-point measurement and the conventional carotid-femoral PWV [20, 27].

The present study is the largest Chinese cohort with longitudinal follow-up data of arterial stiffness. The average value of baseline PWV in the present study (6.1 ± 1.1 m/s) was close to that of one previous report measuring one-point carotid PWV (6.1 ± 1.2 m/s) [19] and was slightly lower than those of previous reports investigating carotid-femoral PWV (7.2–9.7 m/s) in Chinese populations [28, 29]. It appeared that Chinese patients had lower PWV values than Caucasians (8.6–11.40 m/s) [12, 30]. The median value of one-point carotid PWV (5.8 m/s) was systemically lower than that of carotid-femoral PWV (7.2 m/s) [27], possibly explaining why we observed a lower average value. The progression rate of PWV in the Caucasian population varied greatly among studies (0.14 ± 0.22–0.29 ± 0.31 m/s/yr) [15, 30]. Further investigations are needed to validate the progression rate found in our population (0.19 ± 0.20 m/s/yr).

Arterial stiffening is one of the manifestations of vascular aging. The Framingham Heart Study showed that aging is strongly correlated with stiffer arteries [31]. Our longitudinal cohort study further demonstrated that aging is not only associated with arterial stiffness at a cross-sectional time point, but was also related to the acceleration of stiffness progression, in accordance with several other longitudinal studies [11, 13, 32]. In the MESA study, the rate of progression became steeper in subjects older than 75 years old [13]. Similarly, our cohort found that subjects aged ≥ 70 years had significantly faster stiffness progression rates than those in other age groups (Additional file 1: Table S3). In line with previous reports [11, 33], stiffness progression rates were found to be similar between men and women in our population. According to the Baltimore Longitudinal Study of Aging, the progression rates between sexes were different in elderly populations but similar in young subjects [12]. Although men tended to have faster progression rates than women in our subjects aged ≥ 70 years (Fig. 1), a small sample size in this age group limited the statistical power to distinguish whether the difference was substantial.

There was an inverse correlation between the stiffness parameters at baseline and their progression rate at follow-up. In other words, a worse stiffness profile at baseline was associated with a slower progression rate, rather than a faster progression rate. In agreement with our findings, Wildman et al. also found baseline aortic PWV (aPWV) was negatively associated with annual changes in aPWV [33]. Moreover, an inverse correlation was found between baseline carotid intima-media thickness (IMT) and IMT progression in the Young Finns Study [34]. This reverse relationship represents a ceiling effect of arterial stiffness, which indicates that stiffer arteries have less physiological room for further progression [13].

Our data suggested that elevated BP at baseline and during follow-up were two independent predictors of stiffness progression, with increases in BP during follow-up being the stronger of the two (Table 3). Poorly controlled BP was reported to lead to a PWV progression rate that was three times faster in hypertensive patients than in those with well-controlled BP [11]. In our cohort, we found that increases in BP have a deleterious influence on stiffness progression for both hypertensive and normotensive subjects (Fig. 2). This supported the previous findings that pre-hypertension (SBP/DBP = 120-139/80-89 mmHg) as well as clinically defined hypertension were both risk factors for stiffness progression [12, 32]. We also found that the stiffness progression rate was similar between normotensive subjects and treated hypertensive subjects (Additional file 1: Table S4). However, subjects with poorly controlled BP during follow-up had faster stiffness progression whether they were normotensive or hypertensive at baseline. In addition, there was a reciprocal relationship between stiffness parameters and BP. Elevated PWV was found to be a predictor of longitudinal BP changes and incident hypertension [35], while high BP causes blood vessels to lose their elasticity, which in turn makes the control of BP more difficult. Therefore, early intervention to control BP is an important step to maintain the elasticity of blood vessels.

Obesity has been recognized as a risk factor for arterial stiffness in cross-sectional studies [36]. One longitudinal study showed that both baseline BMI and BMI changes were independently associated with an increase in aortic PWV in both men and women [33]. However, our data only suggested that male arterial stiffness was influenced by baseline BMI and BMI changes. Because there was a smaller change of BMI in women relative to men (ΔBMI = 0.32 ± 1.4 in women and 0.48 ± 1.3 kg/m^2^ in men, respectively), it was difficult to observe a significant effect of ΔBMI on stiffness progression in females.

There were several limitations in the present study. We used one-point carotid PWV rather than the gold standard measure of carotid-femoral PWV. Although the two parameters are highly correlated [20, 27], they are not interchangeable, prohibiting head-to-head comparison between our data and other studies. The relationship between medication and the stiffness progression rate was not investigated due to incomplete information concerning medication. However, this study emphasizes the relationship between BP control and stiffness progression, rather than the drug effect of individual anti-hypertensive agents. Although MBP rather than SBP/DBP is used in our regression model, the relationships between BP and stiffness progression are identical for the three BP measurements. We acknowledged that the utilized sample size and duration of follow-up might have limit the power to detect a risk factor with a modest effect, especially for sex-specific analyses. However, our sample size was comparable with previous cohorts [11, 12, 15], and our results are consistent with these previous studies.

## Conclusions

The present study delineated the progression rate of arterial stiffness and predictors of stiffness progression in a Chinese population. Aging and elevated blood pressure at baseline and during follow-up were the major determinants of stiffness progression in this Han Chinese population. For men, increased baseline BMI and changes in BMI were additional risk factors.

## Additional file

Additional file 1: Table S1. The relationship among cardiovascular risk factors, arterial stiffness at baseline, and progression rate. **Table S2.** Relationship between risk factors at baseline and stiffness progression (stratified by sex). **Table S3.** Stiffness progression rates among different age groups. **Table S4.** Stiffness progression rate in normotensive and treated hypertensive patients. (DOC 119 kb)

## Abbreviations

PWV: Pulse wave velocity
EP: Elasticity modulus
β: Arterial stiffness index
MAP: Mean arterial pressure
BMI: Body mass index
BP: Blood pressure
baPWV: Brachial-ankle PWV
KMUH: Kaohsiung Medical University Hospital
TC: Total cholesterol
HDL-C: High density lipoprotein-cholesterol
FBS: Fasting blood sugar
TG: Triglyceride
CCA: Common carotid artery
SD: Standard deviation
aPWV: Aortic pulse wave velocity
IMT: Intima-media thickness
