# Supplementary material for: Determinants of arterial stiffness progression in a Han-Chinese population in Taiwan: a 4-year longitudinal follow-up
Source: BMC Cardiovasc Disord. 2015 Sep 16;15:100. doi: 10.1186/s12872-015-0093-2 (PMC4574178; doi:10.1186/s12872-015-0093-2)
Supplement: Additional file 1: Table S1. — The relationship among cardiovascular risk factors, arterial stiffness at baseline, and progression rate. Table S2. Relationship between risk factors at baseline and stiffness progression (stratified by sex). Table S3. Stiffness progression rates among different age groups. Table S4. Stiffness progression rate in normotensive and treated hypertensive patients. (DOC 119 kb) [file 12872_2015_93_MOESM1_ESM.doc]

**Additional file 1**

**Table S1. The relationship among cardiovascular risk factors, arterial stiffness at baseline, and progression rate**

| **Risk factors at baseline** | **Stiffness parameters at baseline** | | | | **Progression rate of stiffness parameters** | | |
| --- | --- | --- | --- | --- | --- | --- | --- |
| **Hypertension** | (-), n = 407 | | (+), n = 170 | p value | (-), n = 407 | (+), n = 170 | p value |
| PWV | 5.9 ± 1.0 | | 6.7 ± 1.3 | **3.6 x 10^-11^** | 0.19 ± 0.18 | 0.18 ± 0.24 | 0.483 |
| Ep | 95.9 ± 33.7 | | 125.9 ± 55.1 | **3.0 x 10^-10^** | 7.16 ± 7.13 | 8.11 ± 10.93 | 0.295 |
| β | 7.7 ± 2.5 | | 8.9 ± 3.5 | **7.7 x 10^-5^** | 0.64 ± 0.54 | 0.79 ± 0.70 | **0.014** |
| **Diabetes** | (-), n = 524 | | (+), n = 49 | p value | (-), n = 524 | (+), n = 49 | p value |
| PWV | 6.1 ± 1.1 | | 6.5 ± 1.3 | **0.032** | 0.18 ± 0.19 | 0.22 ± 0.24 | 0.264 |
| Ep | 103.8 ± 42. 2 | | 117.3 ± 53.7 | **0.037** | 7.31 ± 8.24 | 8.89 ± 10.10 | 0.216 |
| β | 8.0 ± 2.8 | | 8.9 ± 3.5 | **0.034** | 0.67 ± 0.58 | 0.81 ± 0.68 | 0.110 |
| **Hyperlipidemia** | (-), n = 448 | | (+), n = 125 | p value | (-), n = 448 | (+), n = 125 | p value |
| PWV | 6.1 ± 1.2 | | 6.3 ± 1.0 | 0.126 | 0.19 ± 0.20 | 0.18 ± 0.21 | 0.980 |
| Ep | 103.8 ± 44.9 | | 109.1 ± 37.4 | 0.232 | 7.39 ± 8.35 | 7.60 ± 8.69 | 0.810 |
| β | 8.0 ± 3.0 | | 8.3 ± 2.5 | 0.330 | 0.67 ± 0.58 | 0.72 ± 0.63 | 0.397 |
| **Obesity (BMI ≥ 25)** | (-), n = 335 | (+), n = 179 | | p value | (-), n = 335 | (+), n = 179 | p value |
| PWV | 6.0 ± 1.1 | | 6.5 ± 1.1 | **1.7 x 10^-6^** | 0.19 ± 0.19 | 0.18 ± 0.21 | 0.550 |
| EP | 100.2 ± 41.3 | | 115.2 ± 42.3 | **1.0 x 10^-4^** | 7.36 ± 7.90 | 7.49 ± 8.76 | 0.866 |
| β | 7.8 ± 2.8 | | 8.5 ± 2.9 | **0.005** | 0.67 ± 0.56 | 0.70 ± 0.60 | 0.579 |
| **Smoking** | (-), n = 469 | | (+), n = 94 | p value | (-), n = 399 | (+), n = 72 | p value |
| PWV | 6.2 ± 1.1 | | 6.1 ± 1.1 | 0.498 | 0.18 ± 0.20 | 0.20 ± 0.20 | 0.560 |
| Ep | 105.5 ± 44.6 | | 102.1 ± 37.9 | 0.482 | 7.38 ± 8.43 | 7.59 ± 8.72 | 0.823 |
| β | 8.1 ± 3.0 | | 7.8 ± 2.5 | 0.311 | 0.68 ± 0.59 | 0.68 ± 0.65 | 0.998 |

p value was calculated by Student’s t test.

**Table S2. Relationship between risk factors at baseline and stiffness progression (stratified by sex)**

| **Progression rate** | **Women** | | | **Men** | | |
| --- | --- | --- | --- | --- | --- | --- |
|  | HTN (-), N = 251 | HTN (+), N = 102 | p value | HTN (-), N = 156 | HTN (+), N = 68 | p value |
| ΔPWV/yr | 0.18 ± 0.17 | 0.16 ± 0.25 | 0.453 | 0.20 ± 0.19 | 0.20 ± 0.23 | 0.824 |
| ΔEp/yr | 6.76 ± 6.66 | 7.51 ± 11.30 | 0.533 | 7.80 ± 7.80 | 9.02 ± 10.37 | 0.385 |
| Δβ/yr | 0.62 ± 0.50 | 0.79 ± 0.68 | **0.021** | 0.67 ± 0.59 | 0.78 ± 0.74 | 0.237 |
|  | DM (-), N = 333 | DM (+), N = 17 | p value | DM (-), N = 191 | DM (+), N = 32 | p value |
| ΔPWV/yr | 0.18 ± 0.19 | 0.18 ± 0.26 | 0.927 | 0.19 ± 0.20 | 0.23 ± 0.22 | 0.291 |
| ΔEp/yr | 6.98 ± 8.03 | 7.79 ± 12.44 | 0.694 | 7.88 ± 8.59 | 9.43 ± 8.79 | 0.346 |
| Δβ/yr | 0.67 ± 0.56 | 0.78 ± 0.61 | 0.419 | 0.67 ± 0.62 | 0.83 ± 0.72 | 0.209 |
|  | Lipid (-), N = 277 | Lipid (+), N = 73 | p value | Lipid (-), N = 171 | Lipid (+), N = 52 | p value |
| ΔPWV/yr | 0.18 ± 0.19 | 0.18 ± 0.21 | 0.973 | 0.20 ± 0.20 | 0.20 ± 0.21 | 0.953 |
| ΔEp/yr | 6.91 ± 8.09 | 7.41 ± 8.98 | 0.650 | 8.17 ± 8.72 | 7.87 ± 8.33 | 0.823 |
| Δβ/yr | 0.66 ± 0.54 | 0.73 ± 0.63 | 0.346 | 0.69 ± 0.64 | 0.71 ± 0.64 | 0.832 |
|  | Obesity (-), N = 226 | Obesity (+), N = 86 | p value | Obesity (-), N = 109 | Obesity (+), N = 93 | p value |
| ΔPWV/yr | 0.18 ± 0.19 | 0.17 ± 0.20 | 0.659 | 0.20 ± 0.19 | 0.18 ± 0.21 | 0.534 |
| ΔEp/yr | 7.14 ± 8.10 | 7.27 ± 8.61 | 0.901 | 7.80 ± 7.51 | 7.68 ± 8.94 | 0.919 |
| Δβ/yr | 0.67 ± 0.55 | 0.72 ± 0.57 | 0.459 | 0.68 ± 0.60 | 0.68 ± 0.64 | 0.958 |
|  | Smoker (-), N = 333 | Smoker (+), N = 12 | p value | Smoker (-), N = 136 | Smoker (+), N = 82 | p value |
| ΔPWV/yr | 0.18 ± 0.20 | 0.68 ± 0.57 | 0.971 | 0.20 ± 0.20 | 0.20 ± 0.21 | 0.999 |
| ΔEp/yr | 7.03 ± 8.40 | 6.21 ± 5.88 | 0.738 | 8.23 ± 8.49 | 7.80 ± 9.07 | 0.722 |
| Δβ/yr | 0.68 ± 0.57 | 0.52 ± 0.52 | 0.361 | 0.69 ± 0.63 | 0.70 ± 0.67 | 0.877 |

p value was calculated by Student’s t test

**Table S3. Stiffness progression rates among different age groups.**

|  | **<40 y/o**  **(n=28)** | **40~49 y/o (n=146)** | **50~59 y/o (n=266)** | **60~69 y/o (n=110)** | **≥ 70 y/o**  **(n=27)** | **ANOVA** | **Post-hoc analysis between groups (p values) *** |
| --- | --- | --- | --- | --- | --- | --- | --- |
| **ΔPWV/yr (m/s)** | 0.07 ± 0.14 | 0.18 ± 0.16 | 0.18 ± 0.21 | 0.20 ± 0.22 | 0.26 ± 0.26 | p = 0.011 | 1 vs 5 (0.015) |
| **ΔEp/yr (kPa)** | 2.23 ± 4.25 | 6.44 ± 5.92 | 7.22 ± 8.10 | 8.93 ± 9.92 | 14.31 ± 13.52 | p = 4.3 x 10^-7^ | 1 vs 4 (0.005), 1 vs 5 (7.4 x 10^-6^), 2 vs 5 (3.7 x 10^-4^ ), 3 vs 5 (0.001), |
| **Δβ/yr** | 0.28 ± 0.32 | 0.53 ± 0.44 | 0.66 ± 0.53 | 0.86 ± 0.66 | 1.33 ± 1.00 | p = 7.2 x 10^-14^ | 1 vs 3 (0.019), 1 vs 4 (9.9 x 10^-5^), 1 vs 5 (2.6 x 10^-9^), 2 vs 4 (3.0 x 10^-4^), 2 vs 5 (7.2 x 10^-9^), 3 vs 4 (0.047), 3 vs 5 (1.1 x 10^-6^ ), 4 vs 5 (0.005) |

* Only those with significant difference in post-hoc analysis are listed.

**Table S4. Stiffness progression rate in normotensive and treated hypertensive patients.**

|  | Normotensive (n= 390) | Treated Hypertensive (n = 139) | p value |
| --- | --- | --- | --- |
| ΔPWV/yr | 0.18 ± 0.17 | 0.14 ± 0.24 | 0.066 |
| ΔEP/yr | 6.91± 6.79 | 6.23 ± 9.65 | 0.446 |
| Δβ/yr | 0.63 ± 0.53 | 0.71 ± 0.62 | 0.171 |
| Predictor of PWV progression in multivariate regression | regression coefficient , p value | regression coefficient , p value |  |
| Age | 0.006, p < 0.001 | 0.006, p < 0.001 |  |
| Male | NS | NA |  |
| Baseline PWV (m/s) | -0.061, p < 0.001 | -0.088, p < 0.001 |  |
| MAP (mmHg) | NS | 0.005, p = 0.016 |  |
| BMI (kg/m^2^) | 0.012, p < 0.001 | NS |  |
| ΔMAP (mmHg) | 0.006, p <0.001 | 0.012, p < 0.001 |  |

NS = not significant
